# Phase I trial of systemic intravenous infusion of interleukin-13-*Pseudomonas* exotoxin in patients with metastatic adrenocortical carcinoma

**DOI:** 10.1002/cam4.449

**Published:** 2015-03-13

**Authors:** Yi Liu-Chittenden, Meenu Jain, Parag Kumar, Dhaval Patel, Rachel Aufforth, Vladimir Neychev, Samira Sadowski, Sudheer K Gara, Bharat H Joshi, Candice Cottle-Delisle, Roxanne Merkel, Lily Yang, Markku Miettinen, Raj K Puri, Electron Kebebew

**Affiliations:** 1 Endocrine Oncology Branch, National Cancer Institute, National Institutes of HealthBethesda, Maryland; 2 Clinical Pharmacokinetics Research Laboratory, Clinical Center Pharmacy Department, National Institutes of HealthBethesda, Maryland; 3 Tumor Vaccines and Biotechnology Branch, Division of Cellular and Gene Therapies, Center for Biologics Evaluation and Research, Food and Drug AdministrationSilver Spring, Maryland; 4 Laboratory of Pathology, National Cancer Institute, National Institutes of HealthBethesda, Maryland

**Keywords:** IL-13-PE, maximum-tolerated dose, metastatic adrenocortical carcinoma, pharmacokinetics, Phase I, systemic administration

## Abstract

Adrenocortical carcinoma (ACC) is a rare but lethal malignancy without effective current therapy for metastatic disease. IL-13-PE is a recombinant cytotoxin consisting of human interleukin-13 (IL-13) and a truncated form of *Pseudomonas* exotoxin A (PE). The main objectives of this Phase I dose-escalation trial were to assess the maximum-tolerated dose (MTD), safety, and pharmacokinetics (PK) of IL-13-PE in patients with metastatic ACC. Eligible patients had confirmed IL-13 receptor alpha 2 (IL-13R*α*2) expressions in their tumors. IL-13-PE at dose of 1–2 *μ*g/kg was administered intravenously (IV) on day 1, 3, and 5 in a 4-week cycle. Six patients received 1 *μ*g/kg and two patients received 2 *μ*g/kg of IL-13-PE. Dose-limiting toxicity was observed at 2 *μ*g/kg, at which patients exhibited thrombocytopenia and renal insufficiency without requiring dialysis. PK analysis demonstrated that at MTD, the mean maximum serum concentration (*C*_max_) of IL-13-PE was 21.0 ng/mL, and the terminal half-life of IL-13-PE was 30–39 min. Two (25%) of the eight patients had baseline neutralizing antibodies against PE. Three (75%) of the remaining four tested patients developed neutralizing antibodies against IL-13-PE within 14–28 days of initial treatment. Of the five patients treated at MTD and assessed for response, one patient had stable disease for 5.5 months before disease progression; the others progressed within 1–2 months. In conclusion, systemic IV administration of IL-13-PE is safe at 1 *μ*g/kg. All tested patients developed high levels of neutralizing antibodies during IL-13-PE treatment. Use of strategies for immunodepletion before IL-13-PE treatment should be considered in future trials.

## Introduction

Adrenocortical carcinoma (ACC) is a rare malignancy with an incidence of 0.7–2.0 per million people per year 1–3. It has an overall 5-year relative survival rate of 32–42%, and a median survival of 17–32 months 1,4. The treatment of choice for a localized primary or recurrent ACC is surgical resection. However, patients with recurrent or metastatic disease are rarely curable by surgery alone. Other therapeutic options such as systemic chemotherapy and locoregional radiotherapy have limited impact on survival 5. Thus, identifying new therapeutic targets and strategies are of great importance to the treatment of this malignancy.

Interleukin-13 receptor *α*2 (IL-13R*α*2) is a high-affinity receptor for the Th2-derived cytokine interleukin-13 (IL-13), and is overexpressed in several types of cancers as compared to low or absent expression in embryonic cells and normal tissues 6–11. The high-affinity binding of IL-13 to IL-13R*α*2 signals through a STAT-6-independent AP-1-dependent pathway, which leads to increased transforming growth factor-beta (TGF-*β*) activity 12. We previously reported that IL-13R*α*2 was significantly overexpressed in ACCs as compared to normal adrenocortical tissues and benign adrenocortical tumors 13. Thus, IL-13R*α*2 represents a promising therapeutic target for ACC and other solid malignancies such as pancreatic adenocarcinoma and hepatocellular carcinoma 14,15.

IL-13-PE is a chimeric fusion protein consisting of human IL-13 and a truncated form of *Pseudomonas* exotoxin A (cintredekin besudotox, hIL13-PE38QQR) 16,17. Previous studies have shown that IL-13-PE can bind to IL-13R*α*2 positive tumor cells and is highly cytotoxic to these cells in both *in vitro* and *in vivo* models of multiple malignancies 16,18,19. It has been demonstrated that IL-13R*α*2 positive ACC cell lines were sensitive to IL-13-PE as well 13. Moreover, treatment with IL-13-PE caused tumor regression and prolonged survival in a mouse xenograft model of ACC 13. Several Phase I and II clinical trials and one Phase III clinical trial have been performed to evaluate the safety, tolerability, and efficacy of IL-13-PE using regional delivery of the agent for intracranial malignancies 20–24. Here, we report the first Phase I study of systemic intravenous (IV) administration of IL-13-PE in patients with metastatic ACC.

## Materials and Methods

### Eligibility

Patients with metastatic ACC who failed standard treatments were enrolled. The main eligibility criteria included: age ≥18 years; pathological confirmation of positive IL-13R*α*2 expression in ≥30% of the tumor cells by immunohistochemistry (IHC); measurable disease by response evaluation criteria in solid tumors (RECIST v1.1) at presentation; last dose of chemotherapy or last radiotherapy treatment more than 4 weeks prior to starting IL-13-PE treatment; prior or current mitotane therapy was allowed if patients were on the therapy to control hypercortisolemia, tolerating their dose and did not have a tumor response to treatment; no currently active central nervous system metastasis; Eastern Cooperative Oncology Group (ECOG) performance status at 0–2; good organ function. All patients provided written informed consent. The study (Clinicaltrials.gov: NCT01832974) was approved by our Institutional Review Board and conducted in accordance with Helsinki Declaration and good clinical practice guidelines.

### Study design

This was an open-label Phase I study to assess the maximum-tolerated dose (MTD) of IL-13-PE. The dose-escalation strategy involved three cohorts: six patients to receive 1 *μ*g/kg IL-13-PE, three to six patients to receive 2 *μ*g/kg, and three to six patients to receive 3 *μ*g/kg (Table S1). Treatments for all dose levels were intravenously administered over a 1-h infusion on days 1, 3, and 5 of the first week in each 4-week cycle. Patients were planned to receive four cycles of treatment, but additional cycles were allowed if they had stable disease or partial/complete response. In the following text, detailed treatment date will be referred to as C_D_, with C for Cycle and D for Day; specific patient will be referred to as Pt._.

At each dose, only two patients were on treatment until the first cycle was completed. Additional patients at the same dose were enrolled only when no dose-limiting toxicity (DLT) was observed in those patients. Escalation to the next higher dose was permitted if no more than 1/6 of the previous cohort experienced DLT or the first three patients in subsequent cohorts did not experience DLT. MTD was defined as the highest dose that induces DLT in less than two patients in a cohort of six patients. Adverse events (AEs) were graded according to the Common Terminology Criteria for Adverse Events (CTCAE version 4.0). No intrapatient dose escalation or dose reductions were allowed.

### Evaluation

Blood chemistry and urinalysis were tested within 24 h prior to each treatment and daily during week 1 and then weekly (±2 days). Toxicity evaluation, vital signs, ECOG status, and physical examination were completed within 24 h of each treatment. CT scans of the chest, abdomen and pelvis were used to evaluate the response using RECIST criteria v1.1.

### Pharmaceutical information

IL-13-PE was produced in *Escherichia coli* as described previously under clinical grade drug manufacturing by Insys Therapeutics (Chandler, AZ) 17.

### Pharmacokinetics

Blood samples from patients were collected at 30 min before infusion, and at 0, 5, 15, 30, 60, 90, 120, 180, 240 min, and 24 h after infusion completion on C1D1, C1D3, and C2D1 if treatment was continued. Serum samples were obtained by centrifuging the blood sample tubes at 1940 g for 10 min at 4°C. Serum IL-13-PE concentration was measured in duplicates by enzyme-linked immunosorbent assay (ELISA) using Quantikine Human IL-13 Immunoassay kit from R&D systems, Minneapolis, MN. Purified IL-13-PE was used to produce the standard curve. The lower limit of quantification for this assay was 1.7 ng/mL.

IL-13-PE serum concentration data were analyzed to calculate pharmacokinetics (PK) parameters of apparent elimination half-life (*t*_½_), area under the concentration–time curve from time 0 to the last time point (AUC_last_) and extrapolated to infinity (AUC_0–∞_), clearance (CL), and volume of distribution (*V*_z_) via noncompartmental methods using Phoenix WinNonlin Version 6.4 (Pharsight Corp. Mountainview, CA). AUC was computed using the linear (up)/logarithmic (down) trapezoidal rule with a minimum of four quantifiable concentrations. The only observed values included the *C*_max_ and the corresponding peak times (*T*_max_). Dose-proportionality of AUC and *C*_max_ PK parameters was assessed by determining the geometric mean ratio (GMR) of the two different dose levels.

### Anti-IL-13-PE neutralizing antibody detection

A noncommercial, nonisotopic assay was used to detect neutralizing antibody against IL-13-PE. One thousand U251 cells (Human glioma cell line positive for IL-13R*α*2 expression) in 100 *μ*L of complete RPMI 1640 medium were plated on 96-well black-wall clear-bottom plates and allowed to attach in a 37°C incubator with 5% CO_2_ overnight. Medium composition was as described in Ou, W., et al., 2012 25. On the second day, 50 *μ*L serially diluted patient serum was added in quadruplicates into the U251 cells-coated wells. After 2-h incubation in the cell culture incubator, 50 *μ*L of purified IL-13-PE at 1 ng/mL was added to each well. The plates were then returned to the incubator. Four days later, 20 *μ*L of house-made resazurin was added to each well. Fluorescence of the plates was measured using SpectraMax M5 (VWR Corporate, Altlanta, GA) plate reader (Molecular Devices, Sunnyvale, CA) after 4 h (544 nm for excitation and 590 nm for emission).

### Immunohistochemistry

IHC staining for IL-13R*α*2 was performed with Dako Autostainer (Carpinteria, CA). Primary antibody used was goat anti-IL-13R*α*2 antibody (R&D systems, Minneapolis, MN, 1:1000). The signals were detected by ImmPRESS HRP anti-goat Ig (peroxidase) polymer detection kit (Vector Laboratories, Burlingame, CA), developed by DAB+ (Dako, Carpinteria, CA), and followed by a light hematoxylin counterstain. The staining was semiquantitatively assessed for intensity and percent of positive cells. Negative, weakly positive, and strongly positive controls, selected based on RNA expression data, were included in each experiment. The slides were scanned under an Olympus light microscope (Nikon, Tokyo, Japan) and images were acquired at 20× and 40× magnifications.

## Results

### Patient characteristics and treatment dose

**Figure 1:**
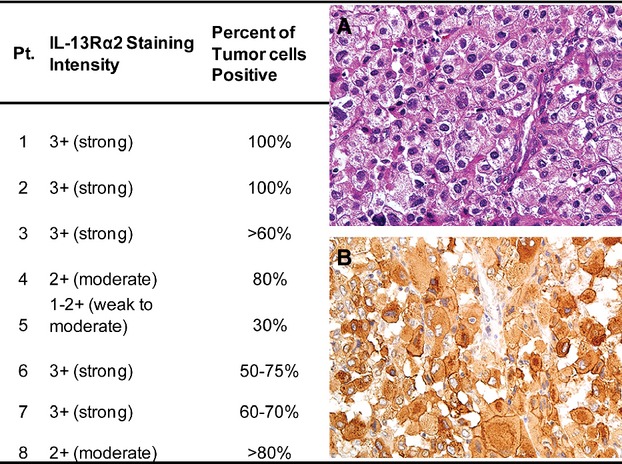
IL-13R*α*2 staining intensity and percent positive cells for each enrolled patient with metastatic ACC. (A) Representative hematoxylin and eosin (H&E) staining of patient tumor. (B) Representative IL-13R*α*2 staining of patient tumor. IL, interleukin; ACC, adrenocortical carcinoma.

Eight patients were enrolled in this study (*N* = 6 at dose 1 *μ*g/kg, *N* = 2 at dose 2 *μ*g/kg) and were evaluable for toxicity. The clinical characteristics of the patients enrolled are summarized in Table1. All patients had stage IV ACC with metastasis to lung (100%), liver (50%) and bone (12.5%). Seven (87.5%) patients had previous operations, two patients (25%) had radiotherapies and all eight (100%) patients had no response to prior systemic chemotherapies. All patients had positive IL-13R*α*2 expression in ≥30% of the tumor cells as determined by IHC (Fig.1).

**Table 1:** Patient characteristics

Demographic and clinical characteristics	*N*	%
Sex		
Male	3	37.5
Female	5	62.5
Age at diagnosis, median (range)	39 (15–64)	
Age at enrollment, median (range)	42 (18–65)	
Body weight in kg, median (range)	82.8 (59.8–125.9)	
Performance status		
0	7	87.5
2	1	12.5
Tumor stage at initial diagnosis		
IV	8	100
Site of tumor at enrollment		
Primary adrenal gland	1	12.5
Adrenal bed (local recurrence)	7	87.5
Regional intra-abdominal recurrence^1^	1	12.5
Lung metastases	8	100
Liver metastases	4	50
Bone metastases	1	12.5
Prior treatment		
Surgery^2^	7	87.5
Radiotherapy	2	25
Chemotherapy	8	100
Mitotane monotherapy	7	87.5
Etoposide, doxorubicin, cisplatin, and mitotane	8	100
Protease inhibitor^3^	1	12.5
Abraxane	1	12.5

1 A single patient was found to have metastatic disease to the intra-abdominal mesentery.

2 Prior surgeries include primary resection of adrenocortical carcinoma, metastectomy, and debulking procedures.

3 A single patient underwent treatment with proteasome inhibitors, bortezomib, and carfilzomib.

### Adverse events

To determine the safety of IL-13-PE, all AEs that were potentially related to the therapy were examined. Of the six patients treated with 1 *μ*g/kg IL-13-PE, the most common AEs included Grade 1 or 2 anemia, proteinuria, increase in alanine aminotransferase (ALT), aspartate aminotransferase (AST), creatinine and fatigue, all of which were seen in three patients. One patient developed Grade 3 hypertension which resolved within 2 days (Table2). Dose escalation to 2 *μ*g/kg resulted in severe AEs in the first two patients enrolled. Grade 3 anemia was observed in both patients, whereas Grade 3 or 4 proteinuria, creatinine increase, acute kidney injury, hyponatremia, neutropenia, pain, and thrombocytopenia were observed in one of two patients. Both patients recovered from these toxicities with supportive care not requiring hemodialysis. Thus, it was determined that 1 *μ*g/kg was the MTD.

**Table 2:** Adverse events of IL-13-PE in patients treated at 1 and 2 *μ*g/kg.

Common toxicity criteria term	1 *μ*g/kg (*N* = 6)		2 *μ*g/kg (*N* = 2)	
	Grade 1 or 2	Grade 3 or 4	Grade 1 or 2	Grade 3 or 4
	*N* (%)	*N* (%)	*N* (%)	*N* (%)
Anemia	3 (50)	0	2 (100)	2 (100)
Proteinuria	3 (50)	0	2 (100)	1 (50)
Alanine aminotransferase increased	2 (33.3)	0	1 (50)	0
Aspartate aminotransferase increased	2 (33.3)	0	1 (50)	0
Creatinine increased	2 (33.3)	0	2 (100)	1 (50)
Fatigue	2 (33.3)	0	1 (50)	0
Alkaline phosphatase increased	1 (16.7)	0	0	0
Chills	1 (16.7)	0	0	0
Cough	1 (16.7)	0	0	0
Dyspnea	1 (16.7)	0	0	0
Edema limbs	1 (16.7)	0	1 (50)	0
Electrocardiogram QT corrected interval prolonged	1 (16.7)	0	0	0
Headache	1 (16.7)	0	1 (50)	0
Hyperkalemia	1 (16.7)	0	0	0
Hypertension	1 (16.7)	1 (16.7)	0	0
Pericardial effusion	1 (16.7)	0	0	0
Weight gain	1 (16.7)	0	1 (50)	0
Acute kidney injury	0	0	2 (100)	1 (50)
Blood bilirubin increased	0	0	1 (50)	0
Bruising	0	0	1 (50)	0
Epistaxis	0	0	1 (50)	0
Fall	0	0	1 (50)	0
Hematuria	0	0	2 (100)	0
Hypoalbuminemia	0	0	2 (100)	0
Hypocalcemia	0	0	1 (50)	0
Hypokalemia	0	0	2 (100)	0
Hypomagnesemia	0	0	1 (50)	0
Hyponatremia	0	0	1 (50)	1 (50)
Neutropenia	0	0	1 (50)	1 (50)
Nausea	0	0	1 (50)	0
Pain	0	0	0	1 (50)
Thrombocytopenia	0	0	2 (100)	1 (50)

IL-13-PE, interleukin-13-*Pseudomonas* exotoxin.

To determine if the DLT was due to a rapid induction of proinflammatory cytokines, we performed a human 30-plex cytokine array using serum samples within 2 days of the initial treatment. The expression levels of 30 common human pro-/anti-inflammatory cytokines were detected simultaneously within each sample to screen for any candidates whose levels were significantly altered. Among the 30 cytokines tested, eight had expression levels that were below the lowest detection limit in all samples; one (RANTES) had an expression level above the highest detection limit in almost all samples. No significant changes were observed in the expression levels of the remaining 21 cytokines that might have contributed to DLT during the treatment course (Fig. S1).

### PK assessment

**Figure 2:**
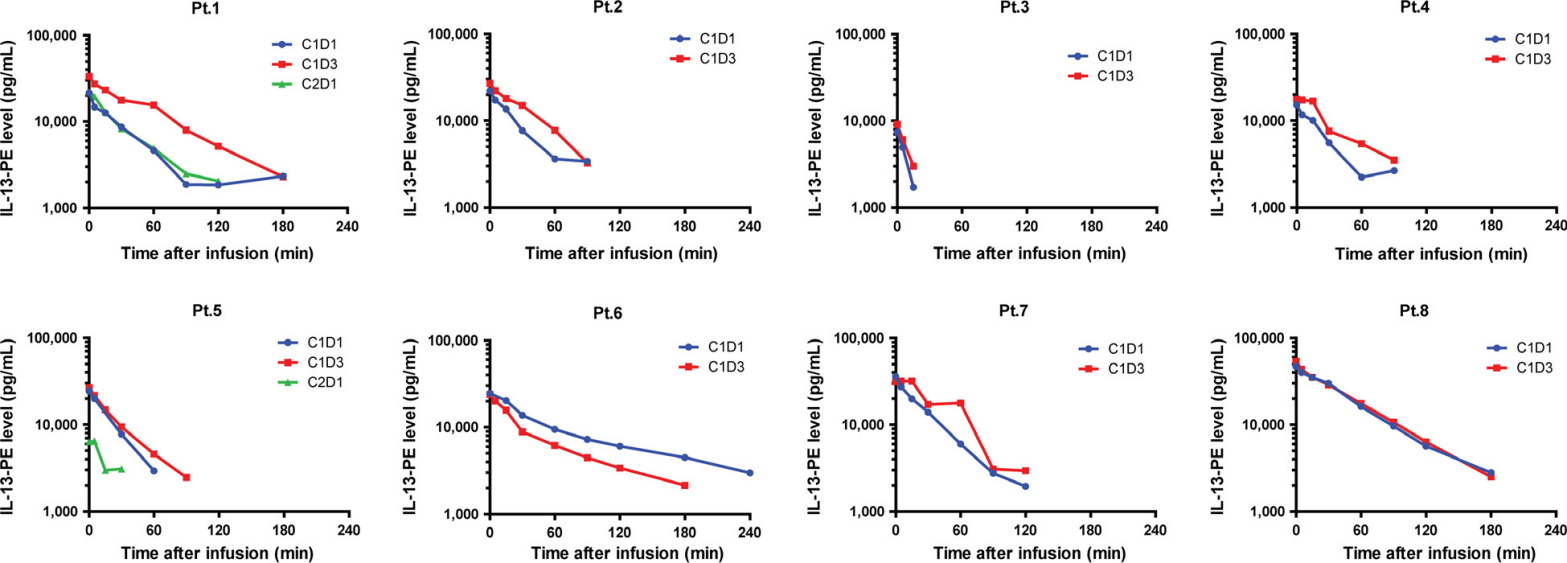
Serum pharmacokinetics of interleukin-13-*Pseudomonas* exotoxin (IL-13-PE) for each patient enrolled. Only serum samples with IL-13-PE concentration above the lower limit of quantification (LLQ) are included. Pt.1 to Pt.6 received 1 *μ*g/kg IL-13-PE, whereas Pt.7 and Pt.8 received 2 *μ*g/kg IL-13-PE.

Serum samples on C1D1 and C1D3 were collected from all eight patients for PK studies, whereas serum samples on C2D1 were collected from Pt.1 and Pt.5. Prior to infusion, none of the patients had a quantifiable level of IL-13-PE in the serum. The time versus serum concentration curves for each patient are shown in Figure2 and the PK parameters related to each dose and treatment cycle are summarized in Table3. After infusion, IL-13-PE serum concentrations declined log-linearly without any significant distribution phase observed. The terminal half-life (*t*_1/2_) was measured to be 30–39 min under all conditions tested. On C1D1, the geometric mean (GM) of *C*_max_ for IL-13-PE was 17.5 ng/mL when treated at 1 *μ*g/kg, and 41.2 ng/mL at 2 *μ*g/kg. After multiple dosing in the first cycle of therapy, no significant accumulation was observed, as the GMRs of AUC_0–∞_ and *C*_max_ (C1D3 vs. C1D1) were 1.23 and 1.20 in the 1 *μ*g/kg dose group and 1.14 and 1.00 in the 2 *μ*g/kg dose group, respectively. In contrast, in the two patients who completed a second cycle of therapy at the 1 *μ*g/kg dose, the GMRs of AUC_0–∞_ and *C*_max_ (C2D1 vs. C1D1) were decreased by 73% and 72%, respectively, in Pt.5, but were not decreased in Pt.1 (Fig.2). The PK of IL-13-PE increased relatively proportionally when the dose was doubled, as the GMRs of AUC_0–∞_ and *C*_max_ (2 *μ*g/kg vs. 1 *μ*g/kg dose groups) were 2.54 and 2.31, respectively.

**Table 3:** Geometric mean (GM) pharmacokinetic parameters of IL-13-PE treatment

Dose	Cycle, day	No. of patients	*C*_max_ (ng/mL)			AUC_last_ (min·ng/mL)			AUC_0–∞_ (min·ng/mL)			% AUC_extrap_		*t*_1/2_ (min)		*V*_z_ (mL)		CL (mL/min)	
			GM	CV	GMR	GM	CV	GMR	GM	CV	GMR	GM	CV	GM	CV	GM	CV	GM	CV
1 *μ*g/kg	C1D1	6	17.5	33.8	NA	1134	57	NA	1262	59	NA	9	36	30	78	3169	26	72	86
	C1D3	6	21	36.6	1.2	1410	51	1.24	1553	49	1.23	9	37	34	55	2917	21	59	82
	C2D1	2	11.7	79.9	0.67	665	92	0.59	836	79	0.66	15	87	36	15	7320	78	142	87
2 *μ*g/kg	C1D1	2	41.2	19	NA	2963	41	NA	3089	41	NA	4	9	38	26	1153	31	21	55
	C1D3	2	41.4	36.1	1	3374	34	1.14	3531	33	1.14	4	33	39	12	1068	37	19	47
1 *μ*g/kg	All days	6	17.9	39.4	NA	1154	56	NA	1300	55	NA	9.8	61	33	60	3447	73	73	87
2 *μ*g/kg	All days	2	41.3	23.8	2.31	3162	31	2.74	3303	31	2.54	4.2	21	38	16	1110	28	20	43.2

IL-13-PE, interleukin-13-*Pseudomonas* exotoxin; *C*_max_, maximum serum concentration; *t*_1/2_, terminal half-life; AUC_last_, area under the curve from time 0 to last quantifiable time point; AUC_0–∞_, area under the curve extrapolated from time 0 to infinity; % AUC_extrap_, percentage AUC extrapolation; *V*_z_, volume of distribution; CL, clearance; CV, coefficient of variation; GMR, geometric mean ratio; NA, not applicable.

### Immunogenicity of IL-13-PE

**Figure 3:**
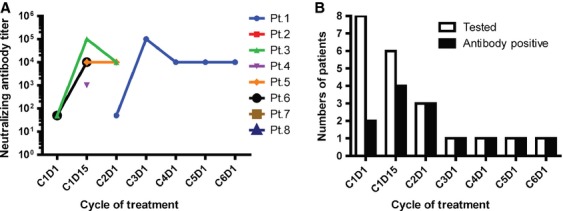
Immunogenicity of interleukin-13-*Pseudomonas* exotoxin (IL-13-PE). The presence of neutralizing antibodies against IL-13-PE was monitored over time. (A) Neutralizing antibody titer detected in each patient during treatment. (B) Numbers of patients tested (Open bar) and positive (black bar) for neutralizing antibodies against PE or IL-13-PE at each treatment cycle.

To determine if neutralizing antibodies against PE were present before treatment or generated against IL-13-PE during treatment, serum samples before each treatment cycle plus serum samples from C1D15 (if available) were collected and tested in a nonisotopic cytotoxicity assay. Interestingly, baseline anti-PE antibodies were detected in 2 (25%) of the eight patients at a low titer (50) prior to initiation of C1 treatment (Fig.3, Table S2). By C1D15, neutralizing antibodies were detected in four (67%) of six patients with available serum samples. These included the two patients who had baseline antibodies and two new patients who developed anti-IL-13-PE antibodies after treatment. At the end of C1 (day 29, immediately prior to C2D1), anti-IL-13-PE antibodies were detected in all three tested patients (100%), one of which developed neutralizing antibodies for the first time. Once the antibodies were generated, they were produced rapidly in large quantities. Within 14–28 days of initial detection, the antibody titer was as high as 10^5^.

### Treatment response

Although this was a Phase I trial, we were able to evaluate response to treatment in five of the six patients who received the 1 *μ*g/kg dose (Table S2). Pt.2 was taken off study due to disease progression before the end of C1, thus the response was not measured. Of the five patients assessed, one (20%, Pt.1) had stable disease for 5.5 months and underwent six cycles of treatment, but disease progressed at the end of C6; two patients (40%, Pt.3 and Pt.5) had stable disease for 2 months, but disease progressed at the end of C2; the other two patients (40%, Pt.4 and Pt.6) had progressive disease after C1.

## Discussion

IL-13-PE represents a novel therapeutic strategy that specifically targets cells overexpressing IL-13R*α*2. Here, we provide the results of the first Phase I clinical trial designed to examine the safety profile and effects of systemic IV administration of IL-13-PE in patients with metastatic ACC overexpressing IL-13R*α*2. Based on our study, IV infusion of 1 *μ*g/kg was determined as the MTD for IL-13-PE. At this dose, the most common adverse effects included low grade anemia, proteinuria, fatigue, and increase in ALT, AST, and creatinine.

The first two patients treated with 2 *μ*g/kg of IL-13-PE developed Grade 3 and 4 toxicities most consistent with thrombotic microangiopathy but only required supportive care. Although a kidney biopsy sample was not obtained to determine thrombotic microangiopathy, this toxicity has been previously observed in Phase I trials of other immunotoxins 26. The self-limited toxicities occurred after completion of the first week of IL-13-PE infusion. The mechanism behind immunotoxin-induced thrombotic microangiopathy is not well understood 26. Several mechanisms have been proposed to explain immunotoxin-mediated thrombotic microangiopathy, including toxin-mediated endothelial damage (off-target and targeted effect) and a general proinflammatory response. We analyzed the cytokine profile in all patients before and after IL-13-PE infusion but observed no appreciable change in proinflammatory cytokine profiles before and after IL-13-PE infusion to support the latter hypothesis. As IL-13R*α*2 protein expression was negative in normal human tissues as examined by IHC in human tissue array (Fig. S2), and IL-13R*α*2 gene expression was also undetected in 14 cases of human activated lymphocytes (with IL-2) and 21 cases of blood lymphocytes (data not shown), it is unlikely that the toxicities exhibited in Pt.7 and Pt.8 were due to off-target effect of IL-13-PE.

As IL-13-PE is a recombinant cytotoxin, which contains domain II and III of PE 16, immunogenicity to this cytotoxin may be expected in patients. Neutralizing antibodies present from prior exposure to PE or produced de novo during treatment represents a challenge for therapy using this chimeric cytotoxin. In this study, we observed that antibodies against PE was present at baseline in 2 (25%) of the eight patients. During IL-13-PE treatment, the generation of high titers of antibodies was fairly rapid (within 14–28 days of treatment initiation) and prevailed in all patients who remained on treatment at MTD. Generation of neutralizing antibody may alter PK and subsequently diminish the efficacy of IL-13-PE. For example, the PK data for C1 and C2 were available for both Pt.1 and Pt.5. In Pt.1 the *C*_max_ and CL were comparable on C1D1 and C2D1. In contrast, the *C*_max_ achieved in Pt.5 was much lower on C2D1 than on C1D1 (Fig.2). This would be consistent with the finding that on C2D1, the anti-IL-13-PE antibody titer was much higher in Pt.5 (10^4^) than in Pt.1 (50) (Fig.3A). High levels of existing anti-IL-13-PE antibodies putatively neutralized most of the administered IL-13-PE during treatment, thus the concentration of free IL-13-PE measured in serum was significantly lower in Pt.5.

PK of IL-13-PE showed overall rapid elimination from serum and apparent linear disposition among drug dose levels of 1 and 2 *μ*g/kg, based on evaluation of *C*_max_ and AUC (drug exposure) on each treatment day and cycle. However, due to limited patient numbers, interpatient variability in weight, age, and other factors that may also influence the PK of IL-13-PE, further investigation is needed in future clinical trials involving IL-13-PE. We also do not know if mitotane therapy could have affected the PK data but this is unlikely given that the patients had normal organ function.

Of the five patients treated at MTD and assessed for response, Pt.1 had stable disease for the longest time (5.5 months) before disease progression. Interestingly, Pt.1 had the highest IL-13R*α*2 expression level in the tumors and the latest development of neutralizing antibodies against IL-13-PE (Figs.1 and 3A). However, the limited number of patients makes it difficult to determine whether patient response would correlate with the expression level of IL-13R*α*2.

In conclusion, this is the first Phase I trial designed to test the safety and effects of systemic IV administration of IL-13-PE in patients with metastatic ACC. Our study demonstrated that IV infusion of IL-13-PE at dose 1 *μ*g/kg is safe and tolerated well by patients when administered every other day for three times during week 1 of a 4-week cycle. However, the generation of neutralizing antibody might hinder the effectiveness of the treatment. In future clinical trials, it would be worthwhile to consider strategies for immunodepletion before IL-13-PE treatment to reduce the development of neutralizing antibodies to this immunotoxin. Alternatively, other IL-13 fusion proteins with no or low immunogenicity should be developed.

## Supporting Information

**Figure S1.** Expression levels of 22 cytokines that were detected in at least one patient using human 30-plex cytokine array. Human Cytokine Magnetic 30-Plex Panel (Life technologies, Frederick, MD) was used to quantitatively determine the level of 30 different cytokines in patient serum. Assay plates were run on Luminex100 platform and data analysis was performed using Bio-Plex manager 6.0 (Bio-Rad, Hercules, CA). Pt.1 to Pt.6 received 1 *µ*g/kg IL-13-PE, whereas Pt.7 and Pt.8 received 2 *µ*g/kg IL-13-PE. Only data within the detection limit of the assay are shown. For RANTES, missing data points were all above the highest detection limit of the assay. For the remaining cytokines, missing data points were all below the lowest detection limit of the assay. No significant changes in cytokine levels were observed that could be associated with DLT. (A) Proinflammatory cytokines. (B) Anti-inflammatory cytokines. Compared to other patients, the cytokine expression profile of Pt.2 was very different. Pt.2 had heavier disease burden than other patients, and the adverse event exhibited in this patient (Grade 3 bone pain, Grade 3 hypokalemia and Grade 2 AST increase) was considered unrelated or unlikely attributed to treatment. Thus, the unusual cytokine levels observed in Pt.2 were unlikely to be associated with toxicity.

**Figure S2.** IL-13R*α*2 IHC staining in normal tissue array. FDA998 normal organ tissue array of human, 47 cases/99 cores (US Biomax, Inc., Rockville, MD) was used to stain for IL-13R*α*2. (A) Array layout: Ceg, Cerebral gray matter; Cew, Cerebral white and/or gray matter; Ceb, Cerebellum tissue; Adr, Adrenal gland; Ova, Ovary; Pan, Pancreas; Par, Parathyroid gland; Hyp, Hypophysis; Tes, Testis; Thr, Thyroid; Bre, Breast; Spl, Spleen; Ton, Tonsil; Thy, Thymus gland; Bon, Bone; Lun, Lung; Hea, Heart; Eso, Esophagus; Sto, Stomach; Sma, Small intestine; Col, Colon; Liv, Liver; Sal, Salivary gland; Kid, Kidney; Pro, Prostate; Ute, Uterine cervix; Str, Striated muscle; Ski, Skin; Ner, Nerve; Mes, Mesothelium; Eye, Eye; Lar, Larynx. Blue background: malignant tumor; light purple background: cancer adjacent normal tissue; others: normal tissue. (B) IL-13R*α*2 staining. All normal tissues were IL-13R*α*2-negative as compared to positive control melanoma sample.

**Table S1.** Study design.

**Table S2.** Treatment dosage, IL-13-PE neutralizing antibody production and response to treatment.
