# Supplementary figures and images for: Phase I trial of systemic intravenous infusion of interleukin-13-Pseudomonas exotoxin in patients with metastatic adrenocortical carcinoma
Source: Cancer Med. 2015 Mar 13;4(7):1060–8. doi: 10.1002/cam4.449 (PMC4529344; doi:10.1002/cam4.449)

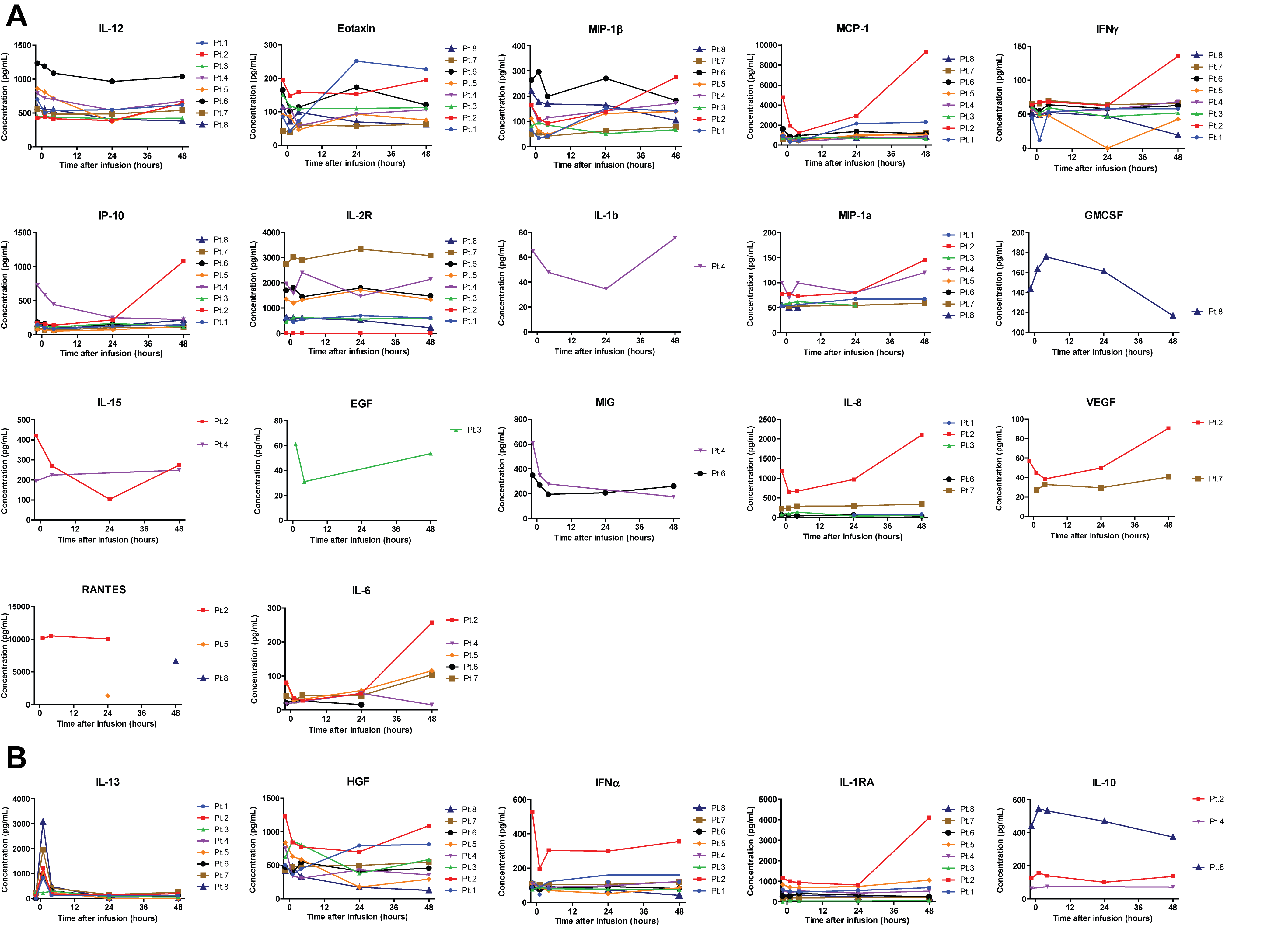

Supplement: Supplementary file 1 [file cam40004-1060-sd1.tif]

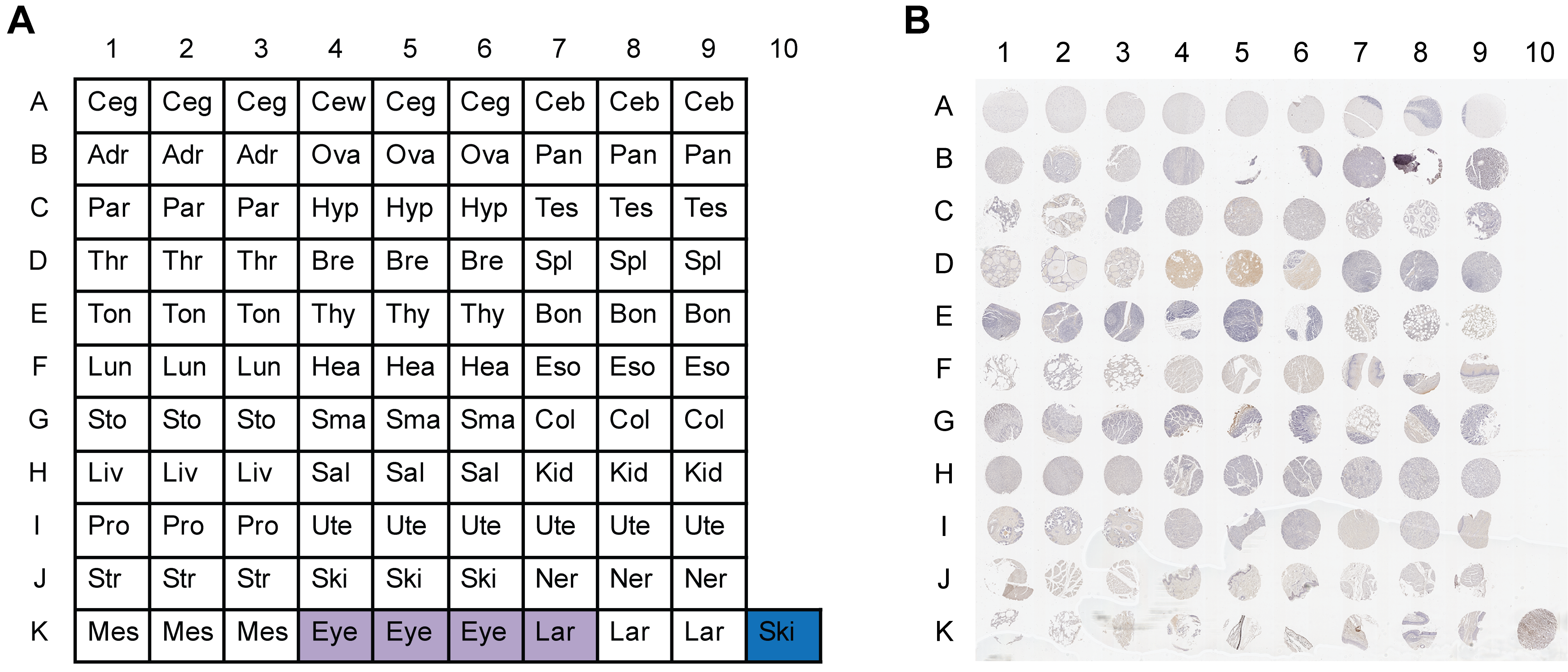

Supplement: Supplementary file 2 [file cam40004-1060-sd2.tif]
