# Supplementary material for: Phase I trial of systemic intravenous infusion of interleukin-13-Pseudomonas exotoxin in patients with metastatic adrenocortical carcinoma
Source: Cancer Med. 2015 Mar 13;4(7):1060–8. doi: 10.1002/cam4.449 (PMC4529344; doi:10.1002/cam4.449)
Supplement: Supplementary file 3 [file cam40004-1060-sd3.docx]

**Supporting Table S1.** Study Design

| Group | 1 Cycle Every 4 Weeks, IL-13-PE Administered on Week 1 of Each Cycle | | | Total Dose | No. of Cycles | No. of Patients to be Accrued |
| --- | --- | --- | --- | --- | --- | --- |
|  | Day 1 | Day 3 | Day 5 |  |  |  |
| A1 | 1µg/kg | 1µg/kg | 1µg/kg | 3µg/kg | <=4^a^ | 6 |
| A2 | 2µg/kg | 2µg/kg | 2µg/kg | 6µg/kg | <=4^a^ | 3-6 |
| A3 | 3µg/kg | 3µg/kg | 3µg/kg | 9µg/kg | <=4^a^ | 3-6 |

^a^ Subject who receives clinical benefit may continue to receive therapy until disease progresses.
