# Supplementary material for: Phase I trial of systemic intravenous infusion of interleukin-13-Pseudomonas exotoxin in patients with metastatic adrenocortical carcinoma
Source: Cancer Med. 2015 Mar 13;4(7):1060–8. doi: 10.1002/cam4.449 (PMC4529344; doi:10.1002/cam4.449)
Supplement: Supplementary file 4 [file cam40004-1060-sd4.docx]

**Supporting Table S2.** Treatment Dosage, IL-13-PE Neutralizing Antibody Production and Response to Treatment

| Patient # | IL-13-PE Dosage | Total Cycles of Treat-ment | Base-line Anti-PE Ab Titer | Date of Last Serum Sample Collected for Ab Detection | Initial Anti-IL-13-PE Ab Detection | | Peak Anti-IL-13-PE Ab Detection | | Response |
| --- | --- | --- | --- | --- | --- | --- | --- | --- | --- |
|  |  |  |  |  | Days since Treatment Initiation | Titer | Days since Treatment Initiation | Titer |  |
| 1 | 1µg/kg | 6 | – | C6D1 | 28 | 50 | 56 | 10^5^ | Stable for 5.5 months then progressed |
| 2 | 1µg/kg | 1 | – | C1D1 |  | – |  |  | Progressed within 1 month |
| 3 | 1µg/kg | 2 | 50 | C2D1 | 0^a^ | 50 | 15 | 10^5^ | Progressed within 2 months |
| 4 | 1µg/kg | 1 | – | C1D15 | 15 | 10^3^ | 15 | 10^3^ | Progressed within 1 month |
| 5 | 1µg/kg | 2 | – | C2D1 | 15 | 10^4^ | 15 | 10^4^ | Progressed within 2 months |
| 6 | 1µg/kg | 1 | 50 | C1D15 | 0^a^ | 50 | 15 | 10^4^ | Progressed within 1 month |
| 7 | 2µg/kg | 1 | – | C1D15 |  | – |  |  | DLT |
| 8 | 2µg/kg | 1 | – | C1D1 |  | – |  |  | DLT |

Abbreviations: Ab, antibody; DLT, dose limiting toxicity.

– Not detected.

^a^ Baseline anti-PE antibody detected before cycle1 treatment.
